# Deletion of immune evasion genes provides an effective vaccine design for tumor-associated herpesviruses

**DOI:** 10.1038/s41541-020-00251-x

**Published:** 2020-11-05

**Authors:** Gurpreet Brar, Nisar A. Farhat, Alisa Sukhina, Alex K. Lam, Yong Hoon Kim, Tiffany Hsu, Leming Tong, Wai Wai Lin, Carl F. Ware, Marcia A. Blackman, Ren Sun, Ting-Ting Wu

**Affiliations:** 1 Department of Molecular and Medical Pharmacology, University of California, Los Angeles, CA 90095 USA; 2 Laboratory of Molecular Immunology, Infectious and Inflammatory Diseases Center, Sanford Burnham Prebys Medical Discovery Institute, 10901 North Torrey Pines Road, La Jolla, CA 92037 USA; 3 Trudeau Institute, Saranac Lake, New York, NY 12983 USA

**Keywords:** Live attenuated vaccines, Herpes virus

## Abstract

Vaccines based on live attenuated viruses often induce broad, multifaceted immune responses. However, they also usually sacrifice immunogenicity for attenuation. It is particularly difficult to elicit an effective vaccine for herpesviruses due to an armament of immune evasion genes and a latent phase. Here, to overcome the limitation of attenuation, we developed a rational herpesvirus vaccine in which viral immune evasion genes were deleted to enhance immunogenicity while also attaining safety. To test this vaccine strategy, we utilized murine gammaherpesvirus-68 (MHV-68) as a proof-of-concept model for the cancer-associated human γ-herpesviruses, Epstein–Barr virus and Kaposi sarcoma-associated herpesvirus. We engineered a recombinant MHV-68 virus by targeted inactivation of viral antagonists of type I interferon (IFN-I) pathway and deletion of the latency locus responsible for persistent infection. This recombinant virus is highly attenuated with no measurable capacity for replication, latency, or persistence in immunocompetent hosts. It stimulates robust innate immunity, differentiates virus-specific memory T cells, and elicits neutralizing antibodies. A single vaccination affords durable protection that blocks the establishment of latency following challenge with the wild type MHV-68 for at least six months post-vaccination. These results provide a framework for effective vaccination against cancer-associated herpesviruses through the elimination of latency and key immune evasion mechanisms from the pathogen.

## Introduction

Human γ-herpesviruses Epstein–Barr virus (EBV) and Kaposi sarcoma-associated herpesvirus (KSHV) are associated with cancer, and with no effective vaccine remain a global health challenge. Despite strong innate and adaptive immune responses, once acquired, herpesviruses persist for the rest of the host’s life. EBV is associated with Burkitt’s lymphoma, nasopharyngeal carcinoma (NPC), and Hodgkin’s- and non-Hodgkin’s lymphomas^1–3^ while KSHV is associated with Kaposi’s sarcoma (KS), primary effusion lymphoma (PEL), and multicentric Castleman’s disease (MCD). These malignancies frequently develop in AIDS patients^4–6^, but also in immunocompetent people, resulting in > 160,000 annual new cancer cases associated with EBV and KSHV^7^. Clearly, effective vaccines against human γ-herpesviruses would dramatically reduce the incidence of malignancies associated with these viruses.

Herpesviruses establish persistent infections characterized by lytic replication and latency. Lytic replication of α- and β-herpesviruses results in disease pathologies, such as varicella and herpes zoster for Varicella-Zoster virus (VZV), cold sores and genital lesions for herpes simplex virus (HSV), and congenital defects for cytomegalovirus (CMV). In comparison, malignancies associated with γ-herpesvirus infection are directly linked to viral latency. Viral genes expressed during latency promote the survival and proliferation of infected cells with increased susceptibility to carcinogenic transformation. Therefore, effective vaccine strategies against tumor-associated herpesviruses ideally should prevent latent infections. The oncogenic potential of γ-herpesviruses has focused vaccine research and development on protein subunit vaccines without the latency risk of live viruses. Subunit anti-EBV vaccines have been based on the envelope protein gp350. Antibodies against gp350 block EBV infection in B-cells^8^ which are long-term latency reservoirs. Gp350-based vaccines protect against infectious mononucleosis (IM); however, they do not influence the overall infection rate^9^ and thus are unlikely to prevent EBV-associated cancers. Similarly, subunit vaccines against HSV-2 may reduce genital lesions but do not prevent infection^10^. Therefore, a new strategy is required to establish wide, durable immunity against herpesviruses.

Live viral vaccines simulate an infection presenting the entire viral antigen repertoire to create stable, long-lasting immune memory. Viruses can be attenuated by removing viral genes essential for replication. However, replication-deficient viruses may undergo recombination and regain replication capacity during propagation in complementing cells expressing the missing genes. Furthermore, attenuation of replication competence may compromise immunogenicity. An alternative approach is to selectively inactivate viral genes involved in immune evasion in order to attenuate replication and enhance immunogenicity. Viral antagonists of type I interferon (IFN-I) response are an important class of immune evasion genes to consider. The IFN-I response is the first line of antiviral defense in the host, and subverting the IFN-I response is critical for viruses to establish infections in hosts. IFN-I response initiates a signaling cascade inducing the transcription of over 300 genes that counteract viral infections^11–14^ and also promotes adaptive immune responses. Approximately 25% of genes encoded by γ-herpesviruses modulate host immunity and include those that counteract the IFN-I response^15,16^.

Here, we designed a viral vaccine that addresses both immunogenicity and safety. We hypothesized that a recombinant herpesvirus lacking multiple IFN-I evasion genes and deficient in latency genes can prime memory development in T and B-cells despite attenuated replication. However, human γ-herpesviruses are highly species-specific and cannot infect small animals. To overcome this limitation, we utilized murine gammaherpesvirus 68 (MHV-68), closely related to EBV and KSHV^17^, to test the hypothesis. We show that an MHV-68 virus engineered to be latency- and immune evasion-deficient is highly attenuated in immunocompetent hosts yet a potent inducer of antiviral immunity. Moreover, this recombinant virus elicits robust long-lasting protection against persistent wild type viral infection.

## Results

### Construction of a virus deficient in immune evasion and persistence (DIP)

In our previous genome-wide screen of MHV-68 open reading frames (ORFs), eight were found to reduce IFN-I responses according to an IFN-stimulated response element (ISRE) reporter assay^18^. We selected *ORF10, ORF36*, and *ORF54* for the insertion of translational stop codons as these genes are dispensable for viral replication and are conserved among MHV-68, KSHV, and EBV. We also inactivated K3, a viral inhibitor of the MHC class I antigen presentation pathway, by truncation to increase the immunogenicity of the vaccine virus^19,20^. We hypothesized that removal of these four immune evasion genes would increase immunogenicity while attenuating replication of the vaccine virus by inducing a robust IFN response and presenting all viral epitopes.

**Fig. 1:**
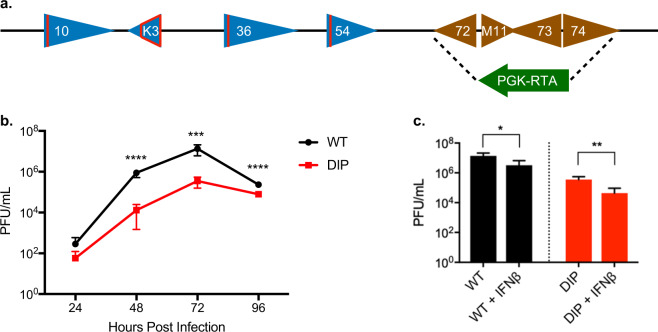
Construction of DIP virus and its replication properties in vitro. **a** Schematic representation of mutations introduced in the MHV-68 genome to generate the DIP vaccine. Red lines indicate insertion of translation stop codons into ORF10, ORF36, and ORF54. The open red tetragon indicates deletion of the coding sequence in K3. The latency locus was replaced by the RTA cassette (arrowhead) constitutively driven by the PGK promoter. **b** Growth curves of the WT and DIP viruses in 3T3 cells using MOI = 0.01 and measured by plaque assay to quantify virion production. **c** NIH 3T3 cells were either mock treated or treated with 100 U mL^−^^1^ IFN-β for 24 h then infected with either WT or DIP virus at MOI = 0.01 for 72 h. Virion production was quantified with plaque assays. All experiments were performed in triplicate and statistical significance was analyzed by a two-tailed Student’s *t*-test. *P* < 0.05*, *P* < 0.01**, *P* < 0.001***, and *P* < 0.0001****. Graphs represent means of triplicates with standard deviations (SD) indicated by error bars.

A critical safety component of our design is eliminating the latency of the vaccine virus. Viral latency is directly linked to tumorigenesis of γ-herpesviruses. Moreover, latent infection also increases the possibility of viral DNA integration into host chromosomes^21^. In KSHV and MHV-68, the biphasic life cycle is regulated by RTA, the replication and transcription activator, and by LANA, the latency associated nuclear antigen. The latter is required for latency establishment^22–24^ while the former upregulates lytic genes^25–27^. We previously showed that abolishing LANA expression combined with constitutive RTA expression results in a latency-deficient virus^28^. Here, we replaced the latency locus comprising ORF72, ORF73 (LANA), ORF74, and M11 with constitutively expressed RTA driven by the phosphoglycerate kinase 1 (PGK) promoter in a two-tiered approach to prevent persistent infection. Deletion of the latency locus, constitutive RTA expression, and the removal of immune evasion genes created a live attenuated γ-herpesvirus vaccine named DIP (deficient in immune evasion and persistence) (Fig. 1a).

### DIP replication is attenuated in vitro

Comparison of the in vitro growth kinetics of DIP in NIH3T3 fibroblasts with the wild type (WT) virus showed that DIP replication was significantly attenuated. After infection at MOI of 0.01, DIP yielded 70-fold and 40-fold less viral production than WT at 48 h and 72 h post-infection, respectively (Fig. 1b). Pretreatment with IFN-β inhibited replication of WT by 4-fold and DIP by 8-fold (Fig. 1c). This larger decrease in infectious DIP virion production confirmed augmented susceptibility to the IFN-I response in the absence of viral IFN evasion genes.

### DIP produces no infectious virions in vivo

**Fig. 2:**
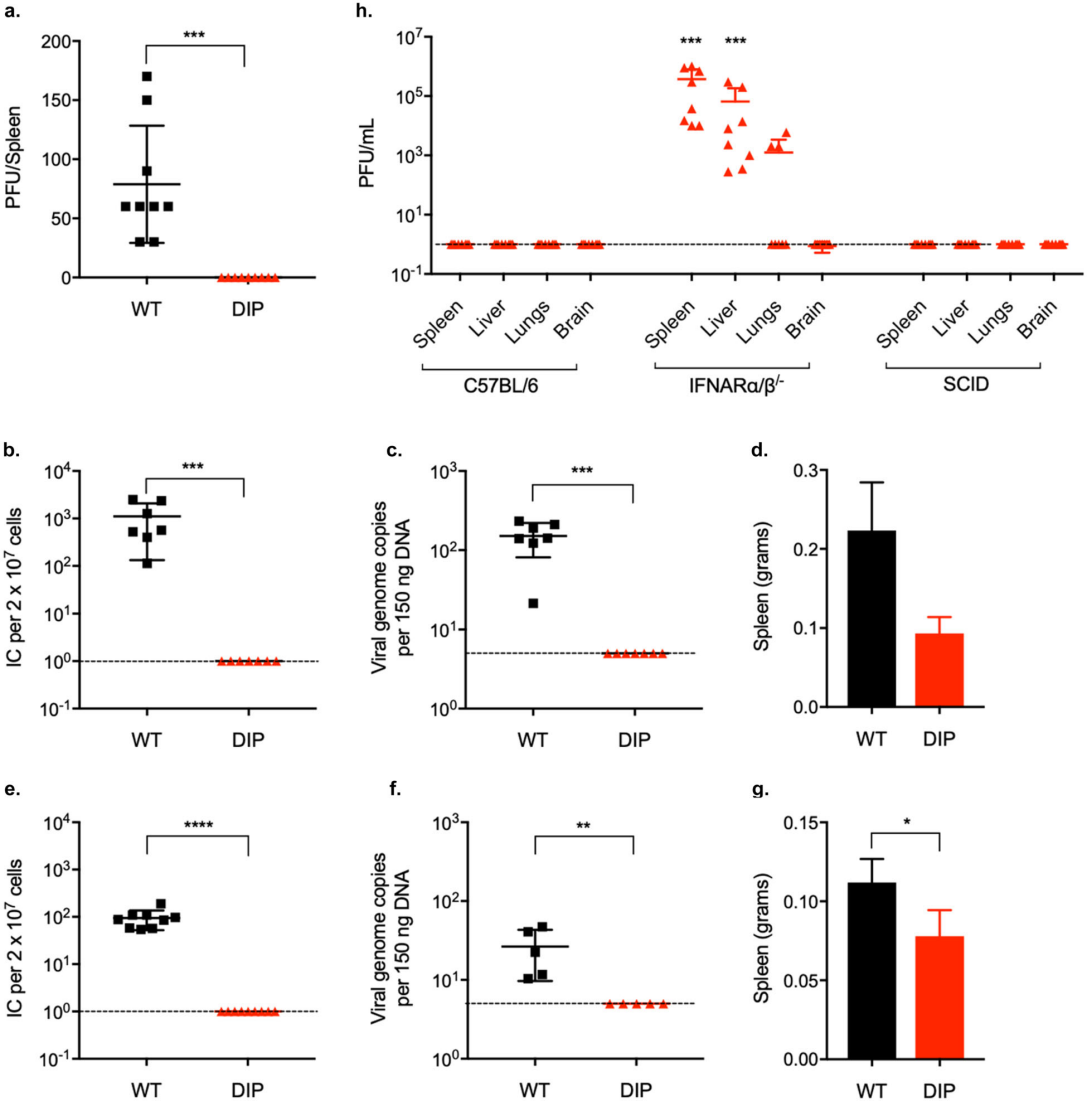
DIP produces no infectious virions and is latency deficient in vivo. All infections were performed intraperitoneally using 10^5^ PFU WT or DIP. **a** Productive infection in the spleens 72 h post-infection was assessed by plaque assay. **b** Latent infection in the spleens at 14 d post-infection was evaluated by infectious center assay and **c** qPCR analysis of viral DNA copy numbers. **d** Spleen weight at 14 d post-infection was measured. No statistically significant difference was found between WT- and DIP-infected mice. **e** Latent infection in the spleen at 2 mo post-infection was measured by infectious center assay and **f** qPCR analysis of viral DNA copy numbers. **g** Spleen weight at 2 mo post-infection was measured. **h** Spleens, livers, lungs, and brains of DIP-infected C57BL/6, IFNARα/β^−^^/−^, and SCID mice were harvested at 3 d post-infection. Infectious viruses were determined by plaque assay. The graphs except **a** depicts the pooled data from 2 independent experiments using different numbers of mice for each replicate. Symbols indicate individual mice and data are means and SD indicated by error bars. Statistical significance was determined by a two-tailed Student’s *t*-test. *P* < 0.05*, *P* < 0.01**, *P* < 0.001***, and *P* < 0.0001****.

We hypothesized that removal of the viral IFN-I evasion genes would generate a highly attenuated vaccine in vivo. To test this, we infected C57Bl/6 mice intraperitoneally and harvested their spleens 3 d after infection. While the WT virus produced 88 PFU/spleen, no infectious virus was detected in the spleens of DIP-inoculated mice (Fig. 2a). We also harvested spleens at later times post-infection and assessed spontaneously reactivating virus by the infectious center assay and quantified latent viral genomes by qPCR. Viral reactivation or latent virus was undetectable in the spleens of DIP-infected mice at 14 d (Fig. 2b, c) and at 2 mo (Fig. 2e, f) after infection. Furthermore, no infectious virion production in the lungs or latency establishment was observed in the spleens after intranasal inoculation (Fig. S1A–D).

Latency establishment is associated with the expansion of Vβ4-specific T-cells and splenomegaly^29–31^. At 14 d post-infection, the spleens of WT-infected mice increased to 0.22 g on average while those of DIP-infected mice weighed 0.10 g (Fig. 2d). The average spleen weight of C57BL/6 J mice is ~0.08 g (http://jackson.jax.org/rs/444-BUH-304/images/physiological_data_000664.pdf). At 2 mo post-infection, WT-infected mice still had significantly larger spleens compared to those of DIP-inoculated mice (Fig. 2g).

To determine whether the IFN-I response contributed to the attenuation of the DIP virus, we injected 10^5^ PFU of DIP intraperitoneally into the interferon-α/β receptor-deficient (IFNARα/β^−^^/−^) mice. DIP replication was rescued and 4 × 10^5^ infectious virions were detected in the spleens at 3 d post-infection (Fig. 2h). Infectious virions were also detected in the livers and lungs but not the brains of IFNARα/β^−^^/−^ mice. In contrast, no detectable infectious virions were recovered from severe combined immune deficiency (SCID) mice. Comprehensive analyses of the spleens, livers, brains, and lungs showed no evidence of infectious virions in either C57BL/6 or SCID mice, both of which have intact IFN-I responses (Fig. 2h).

### DIP immunization prevents latent infection

**Fig. 3:**
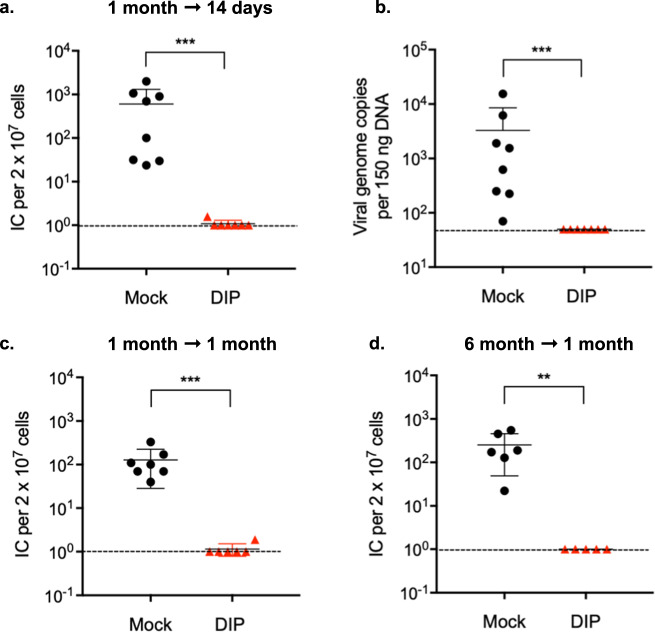
DIP vaccination confers durable protection. Mice were intraperitoneally vaccinated with 10^5^ PFU DIP and challenged intranasally with 5 × 10^3^ PFU WT virus at 1 (**a**–**c**) or 6 (**d**) mo post-vaccination. Latent infection in the spleen was examined at 14 (**a**, **b**) or 28 (**c**, **d**) d after challenge. Viral loads were determined by infectious center assay (**a**, **c**, **d**) and qPCR (**b**). Dotted line indicates detection limit. The graph depicts the pooled data from 2 independent experiments using different numbers of mice for each replicate. Data for individual mice, means, and SD indicated by error bars were plotted. Statistical significance was analyzed by a two-tailed Student’s *t*-test. *P* < 0.05*, *P* < 0.01**, *P* < 0.001***, and *P* < 0.0001****.

Both lytic replication and latent infection contribute to γ-herpesvirus associated malignancies. Moreover, latency is essential for viral persistence and is the main strategy for the virus to avoid elimination by the host immune system^32,33^. Therefore, the goal of vaccination against γ-herpesviruses is to prevent latency establishment. We assessed the level of protection conferred by DIP immunization against latent infection by WT challenge. We previously showed that a replication-deficient MHV-68 virus is much less immunogenic when administered through a mucosal route compared to parenteral administration^34^. While intramuscular injection is the most common parenteral route of administration for human vaccines, due to small muscles in mice, we chose to immunize mice with DIP through an intraperitoneal route to obtain consistent injections. Mice were intraperitoneally injected with 1 × 10^5^ PFU DIP then intranasally challenged 1 mo later with 5,000 PFU WT virus. Mock immunized mice presented an average of 6 × 10^2^ infectious centers per 2 × 10^7^ splenocytes whereas no viral reactivation was detected in six of seven DIP-immunized mice 14 d after challenge (Fig. 3a). Analysis of viral copy number confirmed that DIP immunization provided protection against splenic latent infection (Fig. 3b). At 1 mo post-challenge, five of six (83.3%) DIP-vaccinated mice were completely protected from latent infection (Fig. 3c). The remaining DIP-vaccinated mouse had a 100-fold reduction in latently infected cells compared to mock immunized mice. We also challenged the immunized mice 6 mo after a single vaccination and measured viral latency at 28 d post-challenge. All five DIP-immunized mice were completely protected against latent infection by a WT virus challenge (Fig. 3d).

### DIP primes virus-specific T cells that limit WT infection

**Fig. 4:**
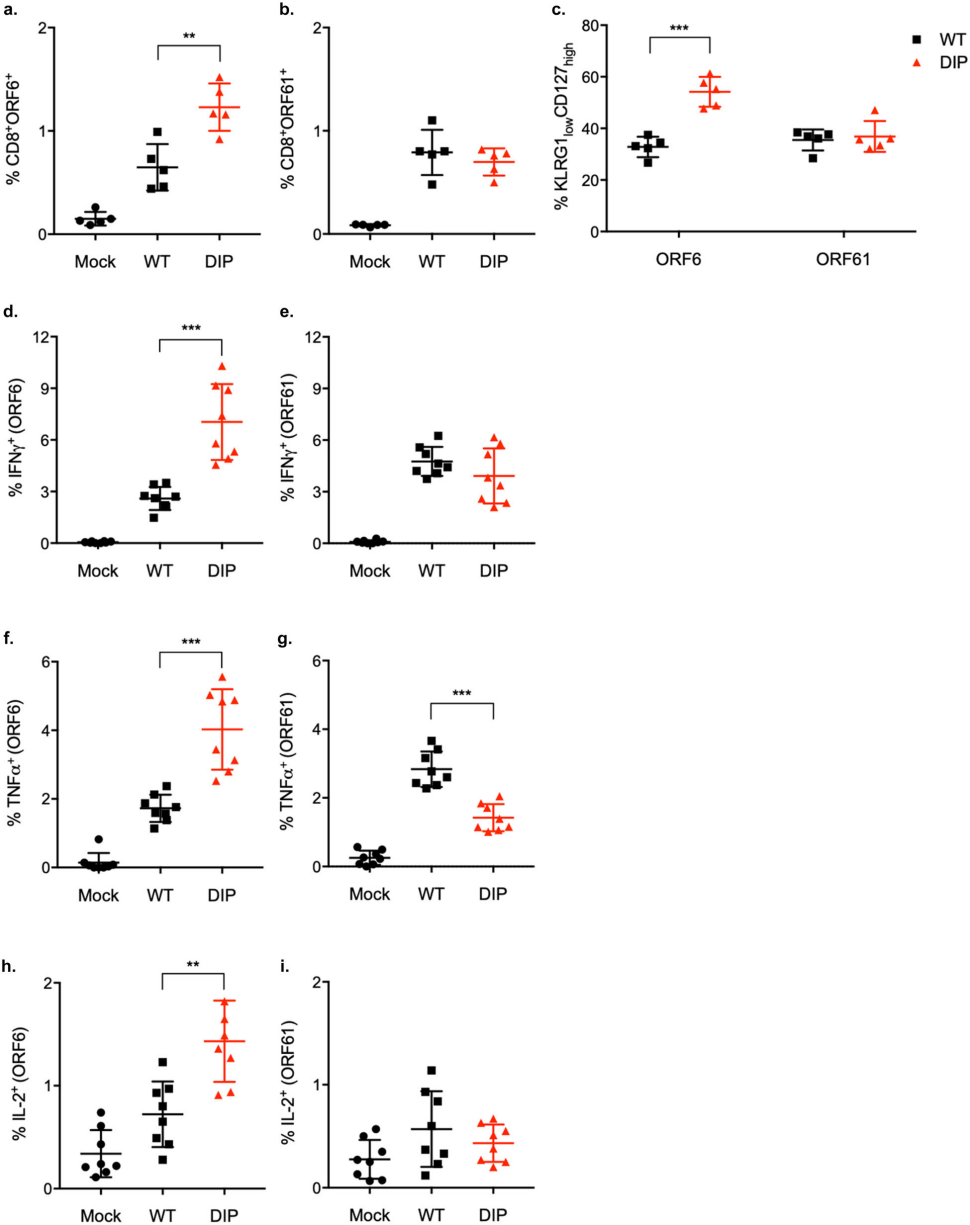
DIP elicits robust virus-specific T-cell immunity. Mice were mock-infected or intraperitoneally injected with 10^5^ PFU WT or DIP. **a, b** At 2 mo post-infection, splenocytes were harvested and examined for virus-specific CD8+ T cells using the tetramers ORF6_487–495_/Db and ORF61_524–531_/Kb. **c** Tetramer-positive CD8+ T cells were examined for KLRG1 and CD127 expression. **d**–**i** Splenocytes were stimulated with ORF6_487–495_ peptide (**d**, **f**, **h**) or ORF61_524–531_ peptide (**e**, **f**, **i**) and stained for intracellular IFN-γ (**d**, **e**), TNF-α (**f**, **g**), and IL-2 (**h**, **i**). Data for individual mice, means, and SD indicated by error bars were plotted. Statistical significance was analyzed by a two-tailed Student’s *t*-test. *P* < 0.05*, *P* < 0.01**, *P* < 0.001***, and *P* < 0.0001****.

We hypothesized that the DIP vaccine elicited a robust and functional T-cell response accounting for the long-lasting protection against WT virus challenge. We quantified virus-specific CD8^+^ T-cells using tetramers for the MHV-68 epitopes, ORF6_487-495_ and ORF61_524-531_. At 1 mo post-infection, WT and DIP-induced similar frequencies of specific T-cells to ORF6 and ORF61 (Supplementary Fig. S2A, B). At 2 mo post-infection, the frequency of ORF6-specific T cells increased two-fold in DIP compared to WT while the frequency of ORF61-specific T-cells were similar (Fig. 4a, b). The effector/memory subtypes of these virus-specific T cells were examined by the expression levels of IL7Rα (CD127) and killer cell lectin-like receptor (KLRG1) (Supplementary Fig. S2D). During the effector phase, the CD127^high^KLRG1^low^ subset consists of memory precursors effector cells (MPECs), which develop into long-lived memory cells, whereas the CD127^low^KLRG1^high^ subset, referred to as short-lived effector T cells (SLECs), are terminally differentiated^35^. The MPEC population after contraction is enriched with memory cells^36^. We observed that DIP promoted the generation of CD8^+^ MPECs. ORF6-specific CD8^+^ T cells (54%) primed by DIP had larger MPEC population compared to those primed by WT (33%). However, no difference was found between WT and DIP infection in terms of ORF61-specific T cells (Fig. 4c and Supplementary Fig. S2C).

We assessed the functions of these virus-specific T-cells by examining their abilities to produce IFN-γ, TNF-α, and IL-2. Consistent with the tetramer-staining results, cells producing IFN-γ, TNF-α, or IL-2 upon stimulation with the ORF6 peptide were twice as frequent in DIP-infected mice as in WT-infected mice (Fig. 4d, f, h). Cells producing IFN-γ upon stimulation with the ORF61 peptide had similar frequencies in WT and DIP-infected mice (Fig. 4e). However, a lower frequency of cells primed by DIP produced TNF-α in response to the ORF61 peptide compared to those primed by WT virus (Fig. 4g). The ORF61 peptide did not stimulate any cells from either WT or DIP-infected mice to produce IL-2 (Fig. 4i). Taken together, despite its limited and transient antigen expression due to highly attenuated replication, DIP still induces robust and functional T-cell responses.

**Fig. 5:**
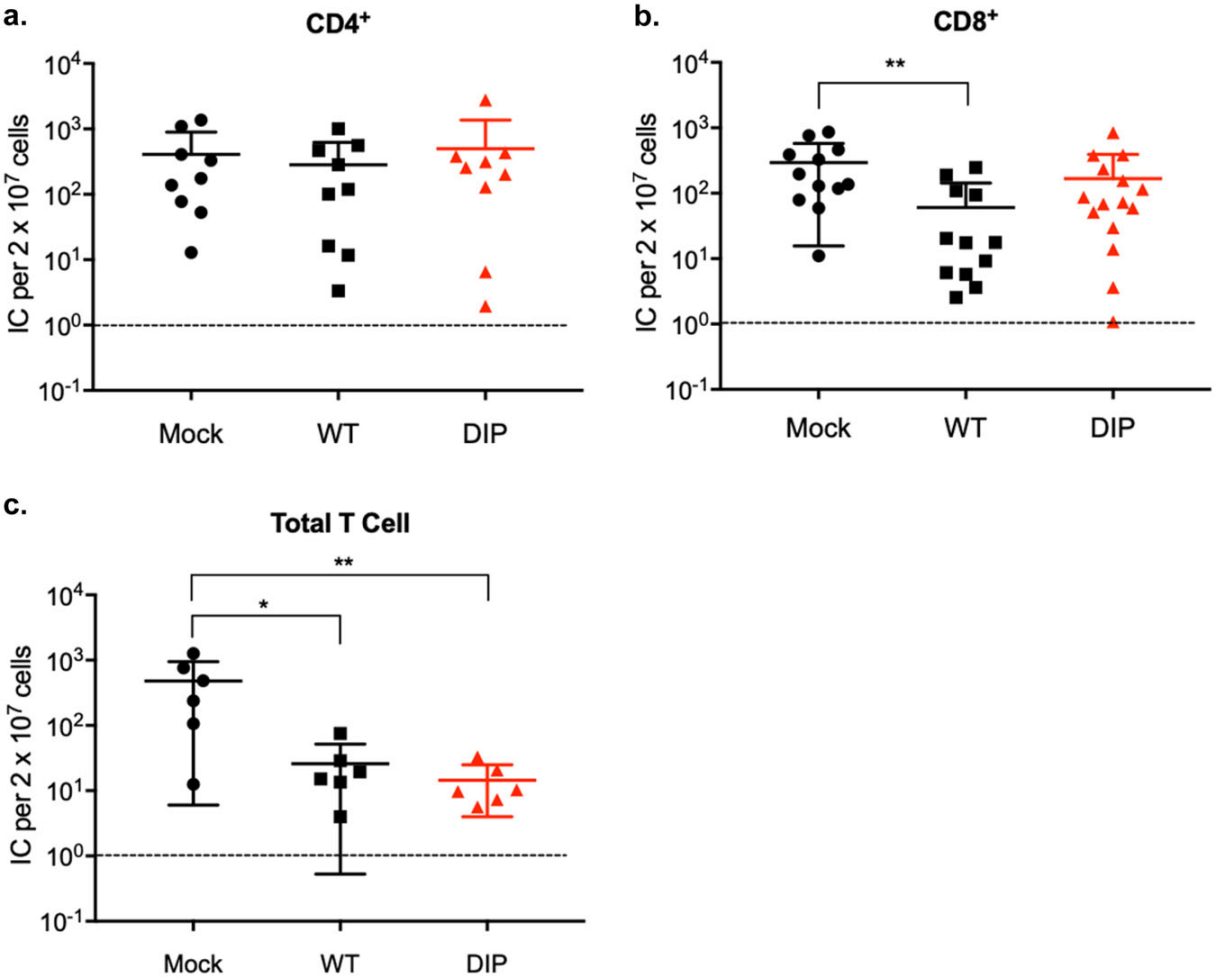
CD4+ and CD8+ T-cells confer antiviral protection. CD4+, CD8+, or total T cells were purified via negative selection from the spleens of mock-infected mice or mice that were intraperitoneally infected with 10^5^ PFU WT or DIP 2 mo previously. Three million CD4+ (**a**), CD8+ (**b**), or total T (**c**) cells were transferred to a congenic mouse by tail vein injection. The recipient mice were intranasally challenged with 5 × 10^3^ PFU WT at 24 h post-transfer. Latent infection in the spleen at 14 d post challenge was measured by infectious center assay. Pooled data from 2 independent experiments using different numbers of mice for each replicate. Data for individual mice, means, and SD indicated by error bars were plotted. Statistical significance was analyzed by a two-tailed Student’s *t*-test. *P* < 0.05*, *P* < 0.01**, *P* < 0.001***, and *P* < 0.0001****.

To determine whether DIP-primed T-cells confer protection against WT challenge, we harvested CD4^+^, CD8^+^ or total T-cells from mock infected mice or mice infected 2 mo earlier with WT or DIP and transferred 3 × 10^6^ of those cells into naïve mice. These recipient mice were then challenged with 5000 PFU WT 1 d after the cell transfer. At 14d post-challenge, we found no significant difference between WT- and DIP-primed T cells in terms of donor cell expansion (Fig. S3). CD4+ T-cell transfer had a minimal impact on the number of latently infected cells (Fig. 5a) despite evidence that CD4+ T cells are cytotoxic to herpesviruses^37–39^. The transfer of WT-primed CD8+ T-cells caused a five-fold reduction in reactivated latently infected cells. In contrast, CD8+ T-cells primed by DIP failed to affect the latently infected cell pool (Fig. 5b). However, mice receiving DIP-primed total T-cells had a 30-fold reduction in the number of reactivated latently infected cells, whereas transferring of WT-primed total T cells caused a 20-fold reduction (Fig. 5c). The results indicate that virus-specific CD4+ and CD8+ T-cells act cooperatively to confer protection. Despite severe attenuation, DIP vaccination elicited robust cellular immunity that inhibits latent infection of the challenge WT virus.

### Optimal DIP-mediated protection requires both antibodies and T cells

**Fig. 6:**
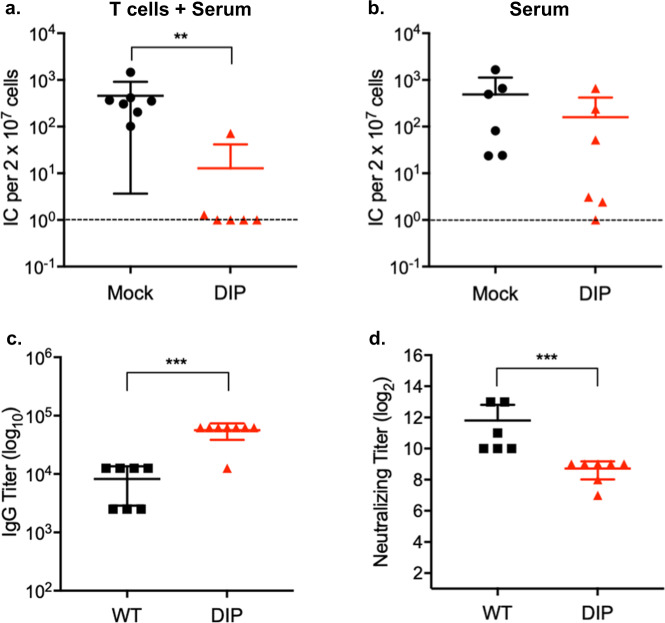
DIP vaccination elicits protective antibodies. Mice were intraperitoneally infected with 10^5^ PFU WT or DIP 2 mo previously. **a** Total T cells and sera isolated from mock- or DIP-infected mice were transferred to congenic naïve mice by tail vein and intraperitoneal injections, respectively. Recipient mice were intranasally challenged 24 h later with 5 × 10^3^ PFU WT virus. Latent infection in the spleen at 14 d post-challenge was assessed by infectious center assay. **b** Sera collected from uninfected- or DIP-infected mice were transferred to naïve mice that were intranasally challenged 24 h later with 5 × 10^3^ PFU WT virus. Latent infection in the spleen at 14 d post-challenge was evaluated by infectious center assay. **c**, **d** Sera collected from infected mice were analyzed for virus-specific IgG by ELISA and for neutralizing activity. Pooled data from 2 independent experiments using different numbers of mice for each replicate. Means and SD indicated by error bars were plotted. Statistical significance was analyzed by a two-tailed Student’s *t*-test. *P* < 0.05*, *P* < 0.01**, *P* < 0.001***, and *P* < 0.0001****.

Although DIP-induced T-cell immunity could reduce latent infection, it did not prevent it (Fig. 5c). Antibodies represent the first line of vaccine-mediated protection. Therefore, to determine whether DIP-elicited antibodies complemented the T-cell-mediated protection, serum and total T-cells from DIP-infected mice were transferred to naïve mice. This combination of T cells and serum completely protected four of six mice against a 5000 PFU WT challenge (Fig. 6a). The two unprotected mice had a significantly reduced number of reactivating latently infected cells compared to the control mice which received serum and T-cells from mock infected mice. We examined the protective capacity of antibodies by passively transferring DIP-immune serum to naïve mice. No significant difference in protection was observed between those receiving DIP-immune serum and those receiving serum from mock infected mice (Fig. 6b). Despite limited effectiveness in vivo by itself the DIP-immune serum contained virus-specific antibodies examined by in vitro assays. Higher levels of virus-specific IgG (Fig. 6c) but yet lower neutralizing activities (Fig. 6d) were detected in the DIP-immune serum than in the WT-immune serum at 2 mo post-infection. Collectively, the data suggest that cellular immunity together with humoral immunity provide optimal protection against latent infection by the challenge WT virus.

### DIP vaccine elicits robust inflammatory responses

**Fig. 7:**
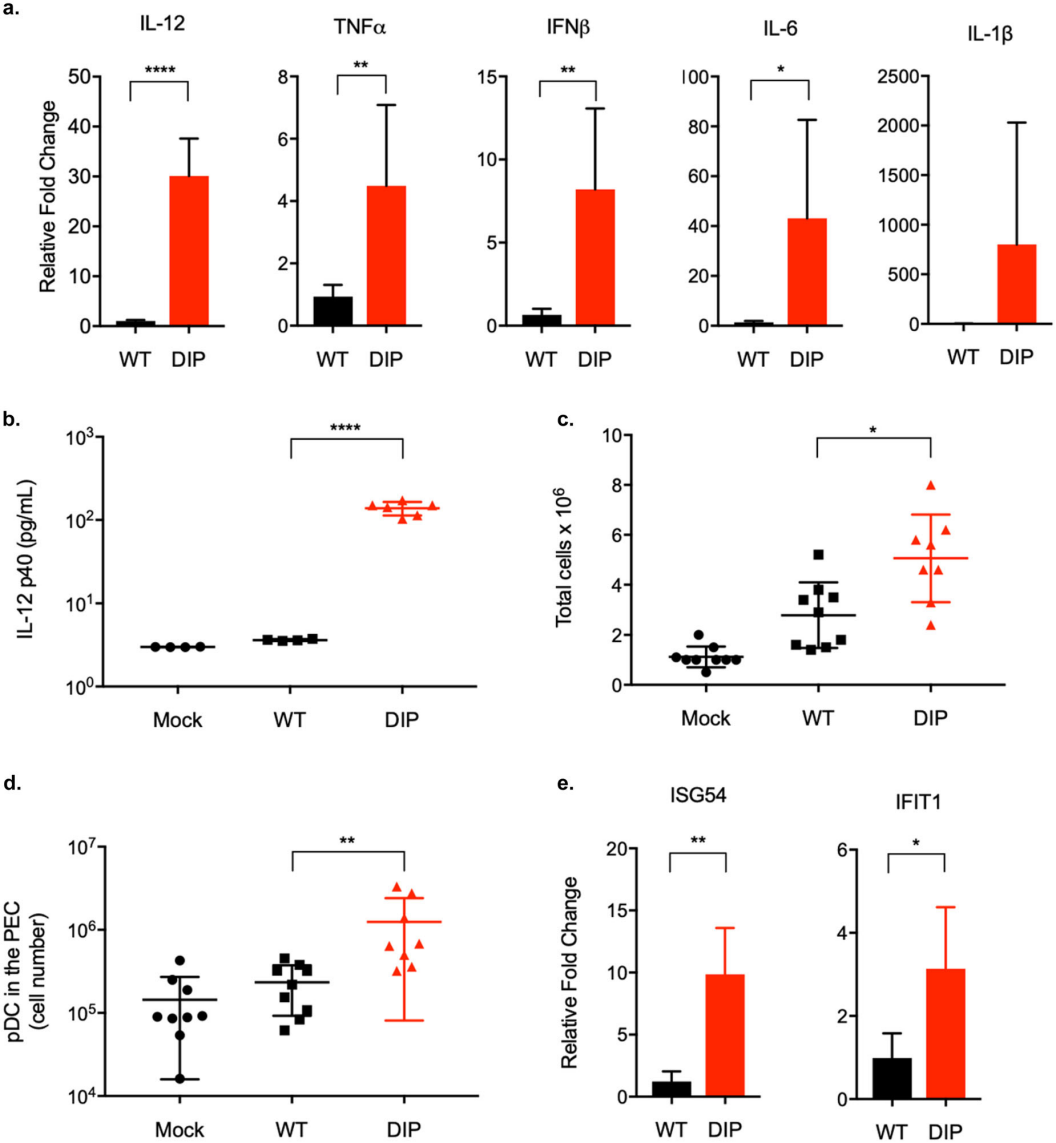
DIP elicits inflammatory and immunomodulatory cytokines. Mouse BMDMs were infected with WT or DIP at MOI = 1 (triplicate). **a** Total RNA was extracted 24 h post-infection for reverse transcription and qPCR to measure the expression levels of IFN-β, IL-1β, TNF-α, IL-6, IL-12, and β-actin. Cytokine RNA expression was normalized against β-actin and the relative fold change was calculated by comparison with mock-infected BMDM. **b** Supernatants were collected 24 h post-infection to measure IL-12p40 production by ELISA. Mice were either mock-infected or intraperitoneally injected with 10^5^ PFU WT or DIP. PECs were collected at 48 h post-infection. **c** Total cell numbers in the PECs were counted. **d** The pDCs were identified by gating on the Lin^-^(CD3^-^CD19^-^NK1.1^-^)B220^+^CD11c^Int^PDCA-1^+^ population. **e** Total RNA was extracted from the PECs. RNA expression of ISGs was analyzed by quantitative PCR. Means and SD indicated by error bars were plotted. Statistical significance was analyzed by a two-tailed Student’s *t*-test. *P* < 0.05*, *P* < 0.01**, *P* < 0.001***, and *P* < 0.0001****.

Despite its limited replication, DIP-primed robust virus-specific immune responses and conferred durable protection. Activation of innate immune response is essential for the development of adaptive immunity. WT MHV-68 avoids inducing inflammatory cytokines in order to evade the immune system. In vitro, a high MOI (100 PFU/cell) was required to elicit a measurable cytokine response in bone marrow-derived macrophages (BMDM) and dendritic cells^40^. We investigated whether DIP induces inflammatory cytokines. BMDMs were infected with WT and DIP virus at MOI of 1 and cytokine RNA expression was quantified at 24 h post-infection. IFN-β, TNF-α, IL-6, and IL-12p40 were significantly upregulated in response to DIP infection compared to WT (Fig. 7a). In addition, WT infection at MOI of 10 still did not induce cytokine expression (Fig. S4). IL-12 is critical for Th1 polarization and cytotoxic cellular immune responses^41^. We validated the IL-12p40 RNA expression by measuring the protein with enzyme-linked immunosorbent assay (ELISA). DIP-induced 30-fold more IL-12p40 protein than WT infection (Fig. 7b). The ability of DIP to stimulate the innate immune responses in vivo was also determined. Two days after intraperitoneal injections of viruses, the average number of peritoneal exudate cells (PECs) in DIP-infected mice was five times the average number in mock-infected mice and significantly higher than in WT-infected mice (Fig. 7c). Flow cytometry analysis of cellular compositions revealed that DIP significantly induced more plasmacytoid DCs (pDCs) than WT virus (Fig. 7d). As pDCs produce IFN-I^42^ and thereby interferon stimulated genes (ISGs), we measured and also detected the upregulation of ISG54 and IFIT2 in the PECs of DIP-infected mice compared to those of WT-infected mice (Fig. 7e). Taken together, the abovementioned results indicate that DIP is highly effective at inducing inflammatory responses.

## Discussion

An effective γ-herpesvirus vaccine should protect against the establishment of latency given its essential role in persistent infection and direct involvement in tumorigenesis. Several vaccine strategies targeting single viral antigens were previously tested in the MHV-68 mouse infection model. These antigens reduced infectious mononucleosis-like symptoms of lymphoproliferation but failed to limit establishment of latency^43–48^. This finding resembles that reported for a clinical trial of EBV gp350-based vaccines^9^. The only vaccine strategy proven to reduce long-term latent viral loads in the MHV-68 model was based on live attenuated viruses designed to be latency-deficient^28,49–53^. However, a major drawback of latency-deficient viruses is the ability to undergo lytic replication^54^. In this study, we tested a strategy to attenuate the in vivo replication of the vaccine virus by inactivating viral antagonists of the IFN-I response. IFN-I is the first line in host antiviral defense and is critical in the development of effective immune responses. IFNs bridge innate and adaptive immunity by activating dendritic cells and inducing Th1 and potent antibody responses^55–58^. IFN-I has been tested as an adjuvant for vaccines against several distinct viruses including influenza, HIV, Ebola, CMV, and γ-herpesviruses^59–63^. Interestingly, Aricò et al. used MHV-68 to demonstrate an increase in viral-specific antibody titers when heat-inactivated virus was co-administered with IFN-α/-β^59^. We proposed that disarming viral IFN-I evasion genes may facilitate the IFN-I response, providing the adjuvanticity required for attenuated viral vaccines. In addition, we also inactivated the viral inhibitor of MHC class I presentation pathway and deleted the latency locus to increase the immunogenicity and safety of the vaccine virus, DIP. The present study demonstrates that DIP is highly attenuated yet maintains overall immunogenicity relatively similar to WT. DIP cannot undergo productive infection or persist in vivo. Despite its attenuated replication, DIP elicits robust innate immune responses (e.g., IL-12), memory T cells, and virus-specific antibodies with neutralizing activity. Single DIP vaccination protected against latency establishment following WT challenge. DIP-mediated protection was durable as all immunized mice remained fully protected even 6 months after a single vaccination.

Antibodies, particularly those that can neutralize infectivity, are the major mechanism by which most licensed prophylactic vaccines provide protection^64–66^. However, for complex pathogens, vaccines may protect through multiple immune mechanisms. Like EBV and KSHV, the B-cell is the main cell type where MHV-68 establishes latent infection^67^. Following intranasal infection, MHV-68 first undergoes acute lytic replication in epithelial cells of the respiratory tract. Subsequently, the virus, likely via trafficking of infected myeloid cells, spreads to secondary lymphoid organs and infects B-cells^68–70^. Viral latency peaks at 2–3 weeks after inoculation and is accompanied by splenomegaly. MHV-68 then establishes a steady, low level persistent infection 4–6 weeks later. Early amplification and long-term maintenance of latency involves viral reactivation to re-infect naïve B-cells^71–73^. This cyclic infection to maintain viral persistence has also been proposed for EBV^74^. Thus, viral lytic replication, particularly in myeloid cells and B-cells, plays a critical role in the establishment of viral latency. This provides the opportunity for the immune responses to control latent infection by targeting lytic antigens, as it has been shown for vaccination against lytic epitopes^44,47,75^. Consistent with this notion, adoptive transfer of DIP-induced T-cells, which should be predominantly specific for lytic antigens, reduced the peak viral latent load (Fig. 5c). On the other hand, although DIP infection elicits neutralizing antibodies, passive transfer of DIP-immune serum alone had little impact on the peak latency (Fig. 6b). A possible explanation for the ineffectiveness of DIP-immune serum is based on the study by Tibbetts et al. that the peak level of MHV-68 latency was comparable over at least a 10^4^-fold range of inoculating doses^76^. Thus, it may be that DIP-induced antibodies failed to completely block infection of incoming virions and that infection of any residual virion would eventually lead to the same peak level of latency. The serum transfer data also imply that DIP-induced antibodies may be unable to control infection of myeloid cells or B-cells during early latency amplification. MHV-68 dissemination within a host, unlike transmissions between hosts, is relatively independent of cell-free virions^77^. However, a supplement of DIP-immune serum to DIP-induced T cells provided a complete protection against viral latency (Fig. 6a). This suggests a synergy between both arms of the adaptive immune response in DIP-mediated protection. It is possible that antibodies reduced the numbers of myeloid cells being infected in the lung to low enough levels enabling T-cells to eliminate the infected cells.

If viruses circumvent antibodies at the entry site of immune hosts and initiate infection, T-cells are required to eliminate virus-infected cells and control dissemination within a host. Naïve CD8+ T-cells only require a brief exposure to antigen to develop effector function and differentiate into memory cells^78–80^. Therefore, despite undetectable replication in vivo, DIP is still capable of inducing memory CD8+ T-cells. However, CD8+ T-cells from DIP-immunized mice were ineffective in reducing viral latent loads compared to those from WT-immunized mice. This may be because CD8+ T-cells from persistently infected mice responded to a secondary challenge more rapidly in producing effectors than those from mice infected with a non-persistent virus^81^. While DIP-induced CD4+ T-cells or CD8+ T-cells alone have little impact on viral latency, they synergize with each other to provide protection (Fig. 5). This CD4-CD8 collaboration occurred with both WT- and DIP-primed T-cells indicating the capability of DIP to elicit effective adaptive immunity. It is recognized that CD4+ T-cells optimize the development and maintenance of memory CD8+ T-cells^82,83^. However, it is unclear whether memory CD8+ T-cells absolutely require this help from CD4+ T-cells or are simply enhanced by them^84^. The expansion of donor CD8+ T-cells in mice receiving total T cells did not surpass that in mice only receiving CD8+ T-cells (Fig. S3). Previous work indicated that memory CD4+ T-cells enhance the functionality of memory CD8+ T-cells^85–87^ but this enhancement was not examined in this study. Furthermore, the effect of memory CD4+ T-cells observed here may not have been mediated by enhancing memory CD8+ T-cells responses. Rather, CD4+ and CD8+ T-cells may target different infected cells, complementing each other, to provide effective protection against latency establishment in response to WT challenge^88,89^. The mechanisms underlying the T-cell collaboration identified herein merit further investigation. It is clear that a prophylactic vaccine against γ-herpesvirus should prime both memory CD8+ and CD4+ T-cells.

A live viral vaccine induces a broad immune response against multiple viral targets. This strategy is particularly advantageous when the mechanisms required for protection against a pathogen are not known. Furthermore, by mimicking an infection, a live vaccine stimulates multiple innate immune responses, robustly induces inflammatory and immunomodulatory cytokines, and provides adjuvanticity for long-lasting vaccine-mediated protective immunity. Nevertheless, most viruses have evolved strategies to counteract the host innate immune system. Data from BMDMs infected in vitro and PECs from infected mice indicates that the DIP virus induces a stronger inflammatory response than WT (Fig. 7), partially accounting for the effective immunogenicity of DIP. DIP also recruits more pDCs than WT, which could also explain the ability of DIP to elicit robust CD8+ T-cells and antibody responses in spite of its highly attenuated replication^90–93^. Future experiments examining the role of specific cytokines or pDCs via genetic knockout and antibody depletion approaches may reveal how DIP-induced innate immune responses impact humoral and cellular immunity. Increased inflammatory cytokine production favors SLEC generation whereas shortening the duration of inflammation has been shown to accelerate MPEC development^81,94^. DIP-mediated inflammatory responses could be short-lived as DIP is highly attenuated in vivo. The heightened but transient DIP-induced inflammation appears to prime a robust T-cell response towards the MPEC phenotype.

In addition to inflammatory cytokines^35,95,96^, duration of antigenic stimulation can also regulate differentiation of effector/memory CD8+ T-cells^97^. Reduced inflammation and shortened antigen exposure promote MPEC differentiation. T-cell epitopes of MHV-68 are differentially presented during infection^98,99^. Some epitopes, represented by ORF6_487-495_, are only presented during the acute lytic replication phase while others, represented by ORF61_524-531_, are continuously presented through the early latency amplification phase. Since ORF6 and ORF61 are both early lytic genes, it is still unclear how presentation of ORF6_487-495_ and ORF61_524-531_ is differentially regulated. Such a difference in epitope presentation may account for the distinct impacts of DIP infection on ORF6- and ORF61-specific cells (Fig. 4). Because of a highly attenuated acute lytic replication, DIP-induced ORF6-specific T-cells should only have a brief exposure to ORF6_487-495_ and cytokines, which favors MPEC differentiation^97^. MPECs that survive the T-cell contraction phase develop into long-lived memory T-cells. As a result, DIP infection leads to a higher frequency of ORF6_487_-specific cells and cytokine-producing T-cells in response to ORF6_487-495_ stimulation (Fig. 4). On the other hand, stimulation with ORF61_524-531_ might continue, perhaps by a specific cell type where only selected lytic antigens, such as ORF61, are expressed and presented, and no preferential differentiation of ORF61_524_-specific cells into MPECs was observed in DIP-infected mice.

Routes of vaccine administrations can affect the cytokine milieus, the type of antigen presentation cells (APCs), and the transport of antigens to lymph nodes. The complexity of various vaccine formulations also makes it difficult to standardize the route of administration^100^. Several human studies showed that in general intradermal injection is more efficient at inducing immune responses than intramuscular and subcutaneous injections reaction^100,101^. Nonetheless, due to a low risk for injection site adverse reactions, intramuscular injection is the most common parenteral route for human vaccines. In this study, intraperitoneal injection was chosen for vaccine administration because in mice it is easy to obtain consistent I.P. injections compared to intramuscular injections. Whether different parenteral routes of administration affect DIP-induced inflammatory responses and subsequent adaptive immunity requires further investigation. Inflammation at the vaccine injection site is essential for recruitment, activation and trafficking of APCs to prime potent adaptive immunity. The ability of DIP to induce robust inflammatory response is likely due to its genetic modifications and should be similar among administration routes.

The development of vaccines against human γ-herpesviruses has been hindered by their restricted host range. Neither EBV or KSHV infects small animals. While the results from mouse studies are not always directly translatable to humans, mouse models have been instrumental in elucidating fundamental principles that cannot be directly tested in humans. MHV-68 infection in mice provides a powerful, easily manipulated small animal model for analyzing fundamental events associated with the infection and immune control of γ-herpesviruses^102–107^. Moreover, the MHV-68 model serves to assess proof-of-concept vaccine strategies^108^. The results from the present study provides the guidance for a rational design of effective live EBV and KSHV vaccines that are highly attenuated and deficient in latency. Deletion of viral immune evasion genes may provide a strategy for the construction of safe yet immunogenic live vaccines against other pathogens.

## Methods

### Viruses and cells

WT MHV-68 was obtained from the American Type Culture Collection (ATCC; Vr1465; Manassas, VA, USA). WT and DIP viruses were propagated in 3T3 and Vero cells and titered by plaque assay. Viruses were concentrated by high-speed centrifugation and resuspended in serum-free Dulbecco’s modified Eagle’s medium (DMEM). Vero cells were cultured in DMEM containing 10% (w/v) fetal bovine serum (FBS) supplemented with penicillin and streptomycin. The 3T3 cells were cultured in DMEM containing 10% (w/v) bovine calf serum (BCS) and 1% penicillin and streptomycin.

### Plaque assay

Each sample was serially diluted tenfold and incubated on Vero cells on 12-well plates in duplicate. The inoculum was removed after 1 h of incubation and the cells were overlaid with 1% (w/v) methylcellulose in DMEM containing 10% (w/v) FBS. Six days post-infection, the cells were fixed with 2% (w/v) crystal violet in 20% (v/v) ethanol. Viral titers were determined by counting plaque numbers. To determine viral titers in the mouse tissues, 1-mL homogenates were prepared in a Dounce homogenizer (Thomas Scientific, Swedesboro, NJ, USA) and used for the plaque assay. Plaques were counted and viral titers in each tissue were expressed in PFU mL^−1^.

### In vitro growth curve

The 3T3 cells were plated on media with or without IFN-β (100 U mL^−1^) for 24 h. Cells were infected at MOI = 0.01 with WT or DIP virus for 1 h at 37 °C. The inoculum was then removed and the cells were washed twice with media before adding fresh media with or without IFN-β (100 U mL^−1^). Cells and supernatant were harvested 24 h, 48 h, and 72 h post-infection for the plaque assay.

### Construction of DIP vaccine

The recA+ *Escherichia coli* GS500 harboring a BAC containing the WT MHV-68 genome was used to construct recombinant MHV-68 by allelic exchange with conjugation-competent *E. coli* GS111 containing the suicide shuttle plasmid pGS284^74–76^. For each recombinant MHV-68, an overlap extension PCR was used to construct the unique shuttle plasmid pGS284 harboring the desired mutation and a ~500-bp flanking region. Sequences upstream of the desired mutation (A fragments) were amplified by AF and AR primers. The downstream sequences (B fragments) were amplified by BF and BR primers using wild type MHV-68 virion DNA as the template. The A and B fragments had >20-bp overlapping sequences. For the subsequent PCR reaction, the A and B fragments were used as templates and amplified by AF and BR primers. The final PCR products were digested with the appropriate enzymes and cloned into pGS284. To screen for the correct mutation, restriction enzyme digestion was performed on the PCR products obtained using the AF and BR primers on the BAC MHV-68 clones. Sequential allelic exchanges were conducted to obtain the final recombinant clone containing all the designed mutations (Fig. 1). After the desired recombinant clone was selected, the MHV-68 BAC was purified and transiently transfected with Lipofectamine^TM^ 2000 into 293T cells with equal amounts of plasmid expressing Cre recombinase to remove the BAC sequence. Three days post transfection, a single viral clone was isolated by limiting dilution. It was then propagated for use in subsequent experiments. The viruses were quantified by plaque assay and limiting dilution.

The ORF36 and ORF54 primers were used to construct the shuttle plasmids^18,109^. Primers used to construct the other shuttle plasmids are listed in Supplementary Table 1. Primers 1–8 were used to construct shuttle plasmids for the stop codon mutation. To construct the shuttle plasmid to replace the latency locus with an RTA expression cassette driven by the Phosphoglycerate kinase 1 (PGK) promoter, four fragments were amplified with primers 9–16, A (ORF72), B (RTA coding sequence and poly A tail), C (PGK promoter), and D (ORF74). A and D fragments were amplified from MHV-68 BAC DNA and B fragment was amplified from pCMVFLAG2-RTA(-49)^51^. The ABCD fused fragment was then generated to be cloned into pGS284.

### Mice

All animal experiments were carried out in compliance with the USDA Animal Welfare Act, the Public Health Service Policy on Humane Care and Use of Laboratory Animals, and the Guide for the Care and Use of Laboratory Animals. The animal studies were approved and regulated by the UCLA Chancellor’s Animal Research Committee. Female C57BL/6J, SCID, and B6.SJL-*Ptprc*^*a*^ *Pepc*^*b*^/BoyJ mice were obtained from Jackson Laboratory, Bar Harbor, ME, USA. IFNARα/β^−/−^ mice were donated by Genhong Cheng at UCLA. Mice aged 6–8 wks were intraperitoneally infected with 10^5^ PFU virus in 200 μL. Intranasal vaccinations and challenges were performed by anesthetizing the mice with isoflurane and administering 20 μL virus dropwise. At the endpoint, mice were euthanized and their tissues were collected in 1 mL DMEM and homogenized with mesh filters and a Dounce homogenizer. Tissue lysates were clarified by centrifugation and used in the plaque assays. Their DNA was extracted with a DNeasy blood and tissue kit (Cat. No. 69504; Qiagen, Hilden, Germany). For the infectious center assay and the flow cytometry study, single-cell suspensions were obtained from the spleens and the red blood corpuscles were lysed in ACK (ammonium-chloride-potassium) buffer.

### Phenotyping virus-specific T cells

Before staining, the splenocytes were incubated with FC block (No. 553142; BD Bioscience, Franklin Lakes, NJ, USA). Tetramers were obtained from the NIH Tetramer Core Facility, Atlanta, GA, USA. Allophycocyanin-conjugated MHCI tetramers specific for the MHV-68 epitopes D^b^/ORF6487–495 (AGPHNDMEI, 1:400 dilution), K^b^/ ORF61524–531 (TSINFVKI, 1:400 dilution), and K^b^/ ORF8_604-612_ (KNYIFEEKL, 1:400 dilution) were incubated with splenocytes for 1 h at room temperature. Surface-staining with the following antibodies was performed by incubation at 4 °C for 30 min: anti-KLRG1 (1:200 dilution, No. 46-5893; eBioscience/Affymetrix, Santa Clara, CA, USA), anti-CD127 (1:200 dilution, No. 17-1273; eBioscience/Affymetrix, Santa Clara, CA, USA), anti-CD8 (1:200 dilution, No. 48-0081; eBioscience/Affymetrix, Santa Clara, CA, USA), anti-CD4 (1:200 dilution, No. 11-0042; eBioscience/Affymetrix, Santa Clara, CA, USA), anti-CD3 (1:200 dilution, No. 25-0031; eBioscience/Affymetrix, Santa Clara, CA, USA), anti-CD44 (1:200 dilution, No. 11-0441; eBioscience/Affymetrix, Santa Clara, CA, USA), anti-CD62L (1:200 dilution, No. 83-0621; eBioscience/Affymetrix, Santa Clara, CA, USA), anti-CCR7 (1:200 dilution, No. 47-1971; eBioscience/Affymetrix, Santa Clara, CA, USA), anti-CD45.1 (1:200 dilution, No. 47-0453; eBioscience/Affymetrix, Santa Clara, CA, USA), and anti-CD45.2 (1:200 dilution, No. 12-0454; eBioscience/Affymetrix, Santa Clara, CA, USA). For intracellular staining, BD Cytofix and Cytoperm (Cat. No. 554714; BD Bioscience, Franklin Lakes, NJ, USA) were used before incubating splenocytes with anti-IFN-γ (1:100 dilution, No. 17-7311; eBioscience/Affymetrix, Santa Clara, CA, USA), anti-TNF-α (1:100 dilution, No. 46-7321; eBioscience/Affymetrix, Santa Clara, CA, USA), and anti-IL-2 (1:100 dilution, No. 25-7021; eBioscience/Affymetrix, Santa Clara, CA, USA) antibodies at 4 °C for 30 min. All samples were fixed in 1% (w/v) paraformaldehyde (PFA). All experiments were analyzed on a SORP BD LSRII analytic flow cytometer (BD Bioscience, Franklin Lakes, NJ, USA). Data were analyzed in FlowJo (FlowJo LLC, Ashland, OR, USA).

### Ex vivo T-cell peptide stimulation

B-cells in splenocytes were depleted by incubation in flasks coated with AffiniPure goat anti-mouse IgG (H + L) (Jackson ImmunoResearch Laboratories Inc., West Grove, PA, USA) for 1 h at 37 °C. B-cell-depleted splenocytes from infected mice (CD45.2+) were incubated with naïve splenocytes (CD45.1+) at a 1:1 ratio in culture media containing 10 U mL^−1^ IL-12, 10 μg mL^−1^ brefeldin A, and 1 μg mL^−1^ peptide for 5 h at 37 °C. Splenocytes were stained and processed for flow cytometry with the indicated tetramers and surface marker antibodies.

### Infectious center assay

Serially diluted splenocytes were plated on a Vero cell monolayer and incubated overnight at 37 °C. The splenocytes were aspirated then washed off by gentle agitation. The Vero cells were overlaid with 1% (w/v) methylcellulose in DMEM containing 10% (w/v) FBS for 6 d before fixing with 2% (w/v) crystal violet in 20% (v/v) ethanol. Infectious centers indicated by plaques were counted.

### Quantitative PCR (qPCR)

The qPCR was performed on MJ Opticon 2 using PerfeCTa Fastmix (Quantabio, Beverly, MA, USA). For the viral genome copy number analysis, 150 ng extracted DNA (~2 × 10^4^ cells) and the primers annealed to the upstream of the ORF6 coding sequence (ORF6: 5’-TGCAGACTCTGAAGTGCTGACT-3’ and 5’-ACGCGACTAGCATGAGGAGAAT-3’) were used.

For the RNA expression analysis, cells were harvested in TRIzol (Thermo Fisher Scientific, Waltham, MA, USA) for RNA extraction according to the recommended protocol. Total RNA was treated with DNAse and used for reverse transcription in a qScript cDNA synthesis kit (Quantabio, Beverly, MA, USA) to generate cDNA for qPCR.

### Gene expression analysis by qPCR

Cell lysates were stored in TRIzol at −80 °C. Isolated RNA was treated with DNase I then used to generate cDNA in a qScript cDNA synthesis kit (Quantabio, Beverly, MA, USA) followed by gene expression analysis with PerfeCTa Fastmix (Quantabio, Beverly, MA, USA). The primers used in qPCR are listed in Supplementary Table 2.

### Infection of BMDM

Cells were harvested from bone marrow and differentiated into macrophages (BMDM) by incubation for 7 d in DMEM containing 20% (w/v) FBS, 5% (w/v) M-CSF, 1% (w/v) penicillin and streptomycin, 1% (w/v) glutamine, and 0.5% (w/v) sodium pyruvate. The BMDMs were infected with WT or DIP at MOI = 1. At 24 h post-infection, total RNA was extracted with TRIzol. Supernatants were collected for analysis in an IL-12/IL-23 p40 (total) mouse uncoated ELISA kit (No. 88-7120-22; Thermo Fisher Scientific, Waltham, MA, USA).

### Neutralizing activity

Twofold serially diluted serum was incubated with 100 PFU WT virus for 1 h at 37 °C. The mixture was plated on a Vero cell monolayer for 1 h at 37 °C then removed. The plate was overlaid with 1% (w/v) methylcellulose in DMEM containing 10% (w/v) FBS for 6 d before fixing with 2% (w/v) crystal violet in 20% (v/v) ethanol. The neutralizing titer was taken as the highest dilution maintaining the ability of the diluted serum to reduce the number of plaques by 50% relative to the virus mixture containing fourfold diluted mock serum.

### Virus-specific IgG ELISA

A 5 μg mL^−1^ WT virion antigen solution coated a 96-well plate which was then incubated overnight at 4 °C. The plate was blocked overnight in PBS containing 1% (w/v) BSA and 0.05% (w/v) Tween-20. The plate was washed twice with PBS-T (PBS containing 0.5% Tween-20). Mouse sera were diluted in ELISA buffer (PBS containing 0.1% BSA and 0.025% Tween-20) and incubated on the plate for 1 h at room temperature. The plate was washed thrice with PBS-T and then once with PBS. A substrate solution consisting of one tablet each of *o*-phenylenediamine and urea hydrogen peroxide (No. P9187; Sigma-Aldrich Corp., St. Louis, MO, USA) in 10 mL ddH_2_O, was added to the plate. The plate was incubated for 30 min in the dark at 4 °C. The reaction was stopped by adding 4 N H_2_SO_4_ and the plate was read at 490 nm and 620 nm. The virus-specific IgG titer was taken as the highest dilution generating signals higher than those of the 1:50 diluted mock serum.

### Serum transfer

Sera were obtained from mice at 2 mo post-infection. Then 200 μL pooled heat-inactivated serum was intraperitoneally injected into naïve mice. After 24 h, the naïve recipient mice were challenged intranasally with 5 × 10^3^ PFU WT. A second dose of 200 μL pooled heat-inactivated serum was intraperitoneally injected 7 d after the WT challenge. Splenocytes were harvested 14 d after the challenge for the infectious center assay.

### Adoptive T-cell transfer

Splenocytes were isolated from mice at 2 mo post-infection and pooled from multiple mice. Splenocytes were negatively selected for CD4, CD8, or total T cells using EasySep isolation kits (Catalog Nos. 19765, 19853, and 19851; STEMCELL Technologies Inc., Vancouver, BC, Canada). Negative selection was confirmed by flow cytometry analysis to >90% purity. Three million cells in 100 μL were injected into the tail vein of each B6.SJL-*Ptprc*^*a*^ *Pepc*^*b*^/BoyJ mouse. Twenty-four hours after T-cell transfer, the recipient mice were intranasally challenged with 5 × 10^3^ PFU WT MHV-68. Spleens were harvested 14 d post-challenge for the infectious center assay and flow cytometry to confirm the presence of donor T-cells in the recipient mice with anti-CD45.1 and anti-CD45.2.

### Statistical analysis

Data are presented as means and their differences were analyzed by a two-tailed unpaired Student’s *t*-test unless otherwise indicated. *P* < 0.05*, *P* < 0.01**, *P* < 0.001***, and *P* < 0.0001****.

### Reporting summary

Further information on research design is available in the Nature Research Life Sciences Reporting Summary linked to this article.

## Supplementary information

Supplementary Information

Reporting Summary Checklist

## Data Availability

The data that were generated and analyzed to support the findings of this study are included in the main text of this published article (and its supplementary information files). Additional data are available from the corresponding author upon reasonable requests.
