# Supplementary material for: Deletion of immune evasion genes provides an effective vaccine design for tumor-associated herpesviruses
Source: NPJ Vaccines. 2020 Nov 5;5:102. doi: 10.1038/s41541-020-00251-x (PMC7644650; doi:10.1038/s41541-020-00251-x)
Supplement: Supplementary file 1 — Supplementary Information [file 41541_2020_251_MOESM1_ESM.pdf]

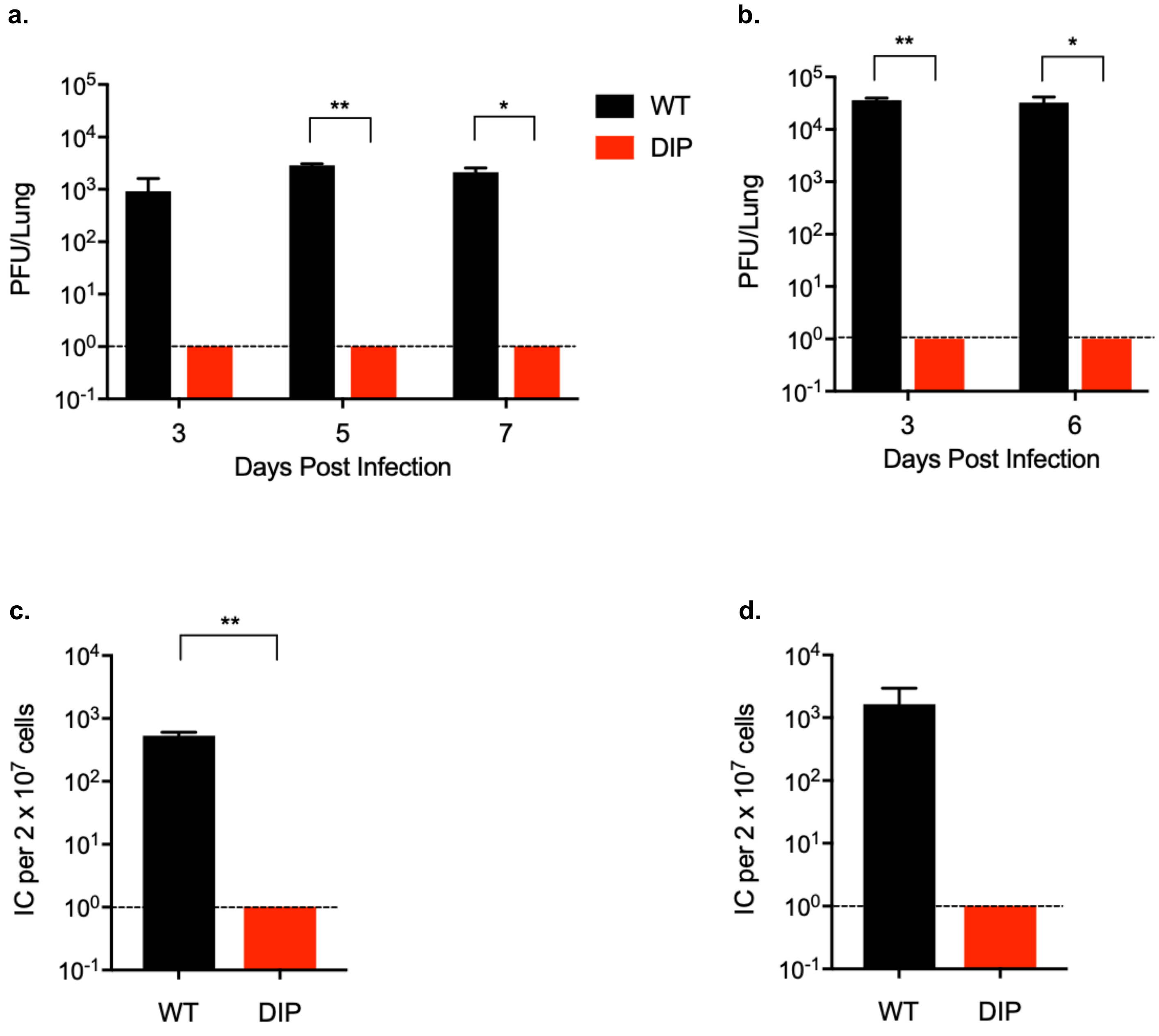

Supplemental Figure S1. In vivo DIP virus is both replication- and latency deficient upon intranasal inoculation. Mice were intranasally inoculated with 5,000 (**a, c**) or 10^5 PFU (**b, d**) WT or DIP. (**a, b**) Lungs (n = 3) were excised at the times indicated at the bottoms of the graphs for plaque assay. (**c, d**) Spleens (n = 3) were excised 14 d post infection for infectious center assay. Means and SD indicated by error bars were plotted. Statistical significance was analyzed by a two-tailed Student's *t*-test. $P < 0.05$*, $P < 0.01$**, $P < 0.001$***, and $P < 0.0001$****.

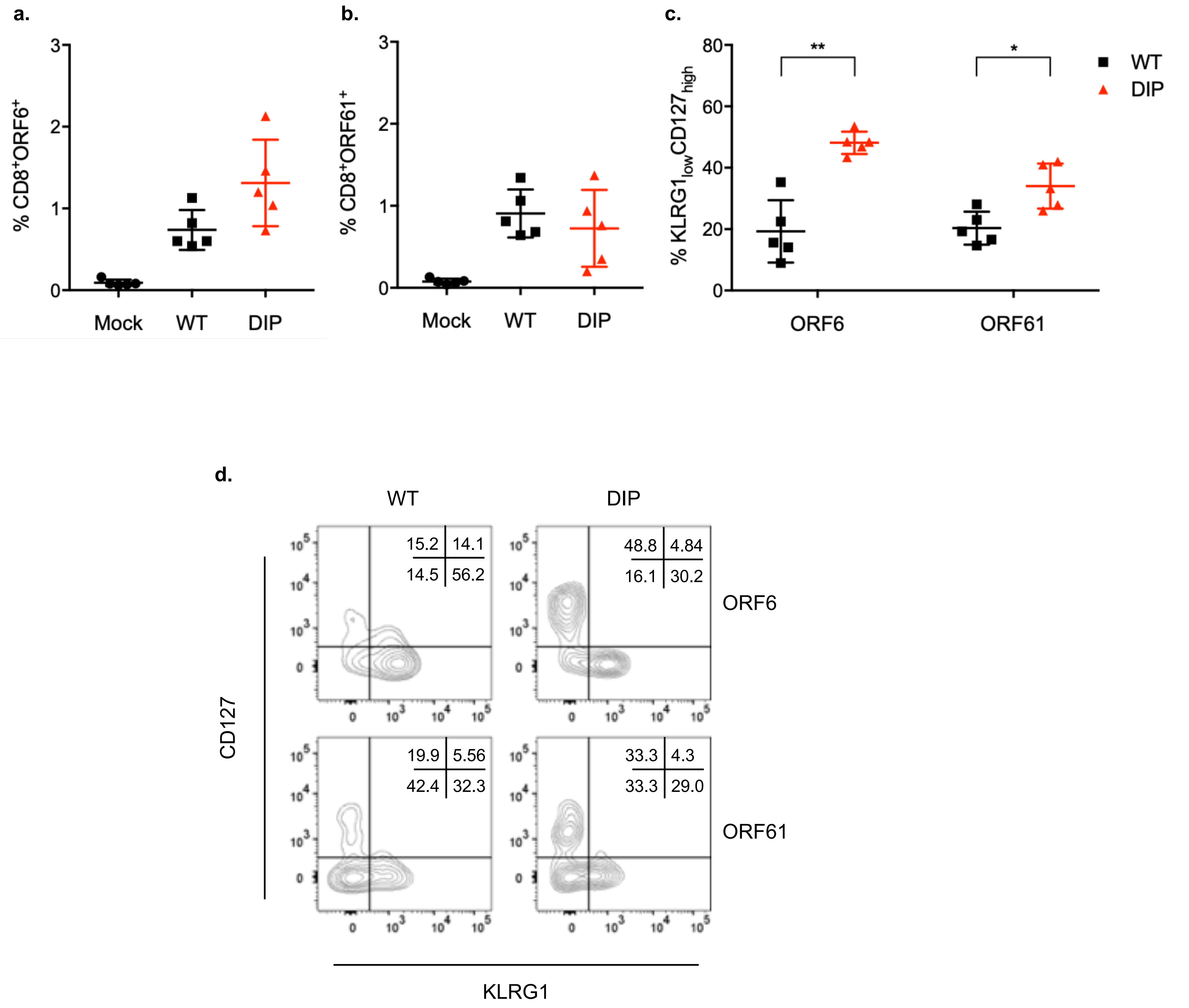

Supplemental Figure S2. DIP infection induces a robust, virus-specific T cell response.
Mice were either mock-infected or intraperitoneally infected with 105 PFU WT or DIP. (**a, b**) At 1 mo post-infection, splenocytes were harvested and examined for virus-specific CD8+ T cells using the tetramers ORF6487–495/Db and ORF61524–531/Kb. (**c**) Tetramer-positive CD8+ T cells were examined for KLRG1 and CD127 expression. Data for individual mice (n = 5), means, and SD indicated by error bars were plotted. Statistical significance was analyzed by a two-tailed Student's t-test. P < 0.05*, P < 0.01**, P < 0.001***, and P < 0.0001****. (**d**) Gating strategy to determine MPEC and SLEC population frequencies using representative samples from WT- and DIP-inoculated mice.

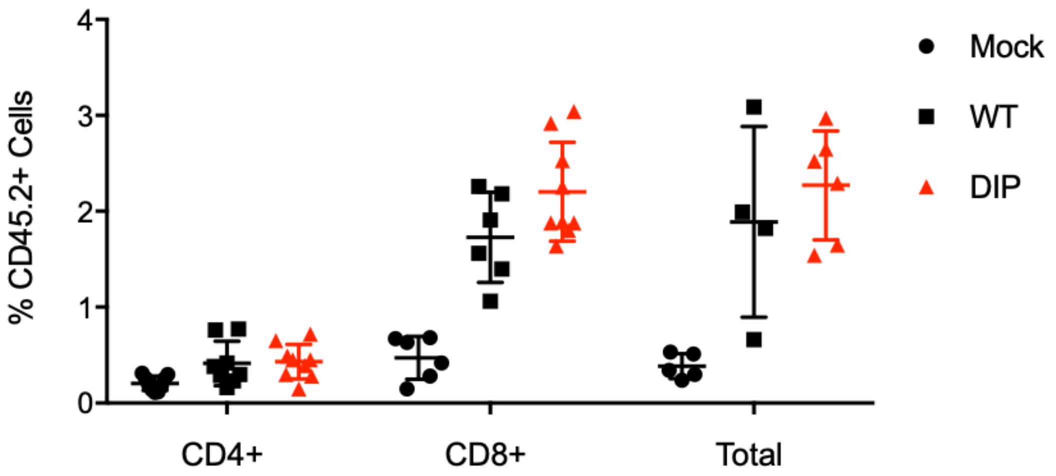

Supplemental Figure S3. WT- and DIP-primed T cells expand to the same extent in the recipient mice.
CD4+, CD8+, or total T cells were purified via negative selection from the spleens of mock-infected mice or mice intraperitoneally infected with 105 PFU WT or DIP. Three million purified cells were transferred to a congenic mouse by tail vein injection. At 14 d after transfer, donor cells were analyzed by flow cytometry and the percentages are shown. Data for individual mice, means, and SD indicated by error bars are plotted. Statistical significance was analyzed by a two-tailed Student's t-test. $P < 0.05$*, $P < 0.01$**, $P < 0.001$***, and $P < 0.0001$****. No statistical significance was detected for the percentages of WT- and DIP-primed donor cells.

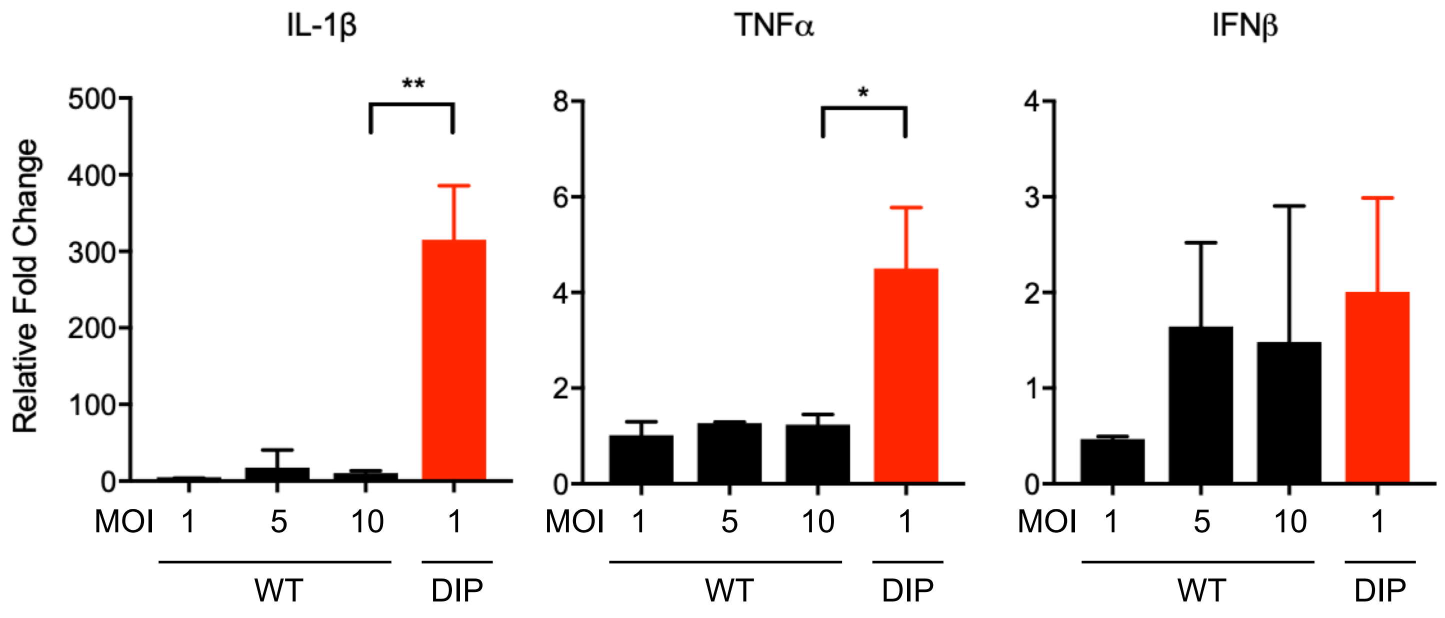

Supplemental Figure S4. WT does not upregulate inflammatory cytokines to the same level as DIP.
Mouse BMDM were infected with WT or DIP at the MOIs indicated at the bottoms of the graphs (triplicate). Total RNAs were extracted 24 h post-infection to measure IFNβ, IL-1β, TNF-α, and β-actin expression. Cytokine RNA expression was normalized against β-actin. Relative fold change was calculated by comparing to mock-infected BMDM. Means and SD indicated by error bars were plotted. Statistical significance was analyzed by a two-tailed Student's t-test. $P < 0.05$*, $P < 0.01$**, $P < 0.001$***, and $P < 0.0001$****.

Supplementary Table 1: Cloning primers

#	Name	Primer Sequence
1	ORF10SAF	ACGCGTCGACCCTCAGATTCATGACAGAATACC
2	ORF10SAR	CAGCTGTCAATTAATTAACCGGGGTGTATCCATTCCTTCTGTTTAG
3	ORF10BF	CCGGTTAATTAATTGACAGCTGACTCTGGACCTACCACTGAC
4	ORF10BR	TTAAGCATGCAGAGACTGATTGGCACAGGCC
5	K3delAF	GCCGAGATCTTCAGGATCTCTGCAACCATG
6	K3delAR	TACTACACTACCGCGGCCGCAAGACTGACCTTGGATTAGC
7	K3delBF	AAGGTCAGTCTTGCGGCCGCGGTAGTGTAGTAGCTCCTGG
8	K3delBR	GCTTGCATGCGGAGAACATATAAGCCTGAC
9	Del72-74+PGKRTA-AF	TCTCGTCGACCTGGTCTAACCGCAAACCGA
10	Del72-74+PGKRTA-AR	CTGCTCCCTTCCCTGTCCTTCCTTTCCTCGTTGCCATAG
11	Del72-74+PGKRTA-BF	CTATGGCAACGAGGAAAGGAAGGACAGGGAAGGGAGCAG
12	Del72-74+PGKRTA-BR	TCATCTCCGGGCCTTTCGAAGAGCTCGTTTAGTGAACC
13	Del72-74+PGKRTA-CF	GGTTCACTAAACGAGCTCTTCGAAAGGCCCGGAGATGA
14	Del72-74+PGKRTA-CR	TTGTTGGGCTAATCTCACACACCGGGTAGGGGAGGCGCTTTTC
15	Del72-74+PGKRTA-DF	GAAAAGCGCCTCCCCTACCCGGTGTGTGAGATTAGCCCAACAA
16	Del72-74+PGKRTA-DR	TCTCGCTAGCGGAAGGGCTACTGCTATGCT

Supplementary Table 2: RT-qPCR primers

Gene	Primer Sequence
Actin	5'-GTATCCTGACCCTGAAGTACC-3'<br>5'-TGAAGGTCTCAAACATGATCT-3'
IL-6	5'-CTGCAAGAGACTTCCATCCAG-3'<br>5'-AGTGGTATAGACAGGTCTGTT-3'
IFNβ	5'-CAGCTCCAAGAAAGGACGAAC-3'<br>5'-GGGAGTGTAACTCTTCTGCAT-3'
IL-1β	5'-TGTTCCTGAACTCAACTGTG-3'<br>5'-GGTCAAAGGTTTGGAAGCAG-3'
IL-12b	5'-TGGTTTGCCATCGTTTTGCTG-3'<br>5'-ACAGGTGAGGTTCACTGTTCC-3'
TNFa	5'-AAATTCGAGTGACAAGCCTG-3'<br>5'-ACCACTAGTTGGTTGTCTTTGAG-3'
ISG54	5'-GGAGAGCAATCTGCGACAG-3'<br>5'-GCTGCCTCATTTAGACCTCTG-3'
IFIT1	5-TGCTTTGCGAAGGCTCTGAAA-3’<br>5’-TTCTGGATTTAACCGGACAGC-3’
