# Supplementary material for: Deletion of immune evasion genes provides an effective vaccine design for tumor-associated herpesviruses
Source: NPJ Vaccines. 2020 Nov 5;5:102. doi: 10.1038/s41541-020-00251-x (PMC7644650; doi:10.1038/s41541-020-00251-x)
Supplement: Supplementary file 2 — Reporting Summary Checklist [file 41541_2020_251_MOESM2_ESM.pdf]

nature research

Corresponding author(s): Ting-Ting Wu

Last updated by author(s): 2020-7-29

Reporting Summary

Nature Research wishes to improve the reproducibility of the work that we publish. This form provides structure for consistency and transparency in reporting. For further information on Nature Research policies, see our Editorial Policies and the Editorial Policy Checklist.

Statistics

For all statistical analyses, confirm that the following items are present in the figure legend, table legend, main text, or Methods section.

n/a	Confirmed	
☐	☒	The exact sample size (*n*) for each experimental group/condition, given as a discrete number and unit of measurement
☐	☒	A statement on whether measurements were taken from distinct samples or whether the same sample was measured repeatedly
☐	☒	The statistical test(s) used AND whether they are one- or two-sided<br>*Only common tests should be described solely by name; describe more complex techniques in the Methods section.*
☒	☐	A description of all covariates tested
☒	☐	A description of any assumptions or corrections, such as tests of normality and adjustment for multiple comparisons
☐	☒	A full description of the statistical parameters including central tendency (e.g. means) or other basic estimates (e.g. regression coefficient) AND variation (e.g. standard deviation) or associated estimates of uncertainty (e.g. confidence intervals)
☐	☒	For null hypothesis testing, the test statistic (e.g. *F*, *t*, *r*) with confidence intervals, effect sizes, degrees of freedom and *P* value noted<br>*Give P values as exact values whenever suitable.*
☒	☐	For Bayesian analysis, information on the choice of priors and Markov chain Monte Carlo settings
☒	☐	For hierarchical and complex designs, identification of the appropriate level for tests and full reporting of outcomes
☒	☐	Estimates of effect sizes (e.g. Cohen's *d*, Pearson's *r*), indicating how they were calculated

Our web collection on statistics for biologists contains articles on many of the points above.

Software and code

Policy information about availability of computer code

Data collection	No software was used in this study.
Data analysis	GraphPadPrism 7 was used for data analysis.

For manuscripts utilizing custom algorithms or software that are central to the research but not yet described in published literature, software must be made available to editors and reviewers. We strongly encourage code deposition in a community repository (e.g. GitHub). See the Nature Research guidelines for submitting code & software for further information.

Data

Policy information about availability of data

All manuscripts must include a data availability statement. This statement should provide the following information, where applicable:

- Accession codes, unique identifiers, or web links for publicly available datasets
- A list of figures that have associated raw data
- A description of any restrictions on data availability

The data that support the findings of this study are available from the corresponding author upon reasonable request.

Field-specific reporting

Please select the one below that is the best fit for your research. If you are not sure, read the appropriate sections before making your selection.

☒ Life sciences ☐ Behavioural & social sciences ☐ Ecological, evolutionary & environmental sciences

For a reference copy of the document with all sections, see nature.com/documents/nr-reporting-summary-flat.pdf

Life sciences study design

All studies must disclose on these points even when the disclosure is negative.

Sample size	Determination of sample sizes were based on previous experience with the model system and pilot experiments to ascertain experimental variance.
Data exclusions	We did not exclude data in this study.
Replication	For the majority of experiments, we performed two independent experiments and included all the data points without exclusion. The data shown in Fig. 4A-C was from one experiment but a similar trend was observed at a different time point and shown in Fig. S2A-C. The data in Fig. 7B was from one experiment but performed in triplicate.
Randomization	Randomization was not applied here.
Blinding	The investigators were not blinded to group allocation during data collection and analysis. This study compared the mutant to the wild type. The phenotypes of the mutant were markedly different from the wild type, which has been thoroughly studied by us and others. Even without the blinding, it was straightforward to distinguish the mutant from the wild type.

Reporting for specific materials, systems and methods

We require information from authors about some types of materials, experimental systems and methods used in many studies. Here, indicate whether each material, system or method listed is relevant to your study. If you are not sure if a list item applies to your research, read the appropriate section before selecting a response.

Materials & experimental systems

n/a	Involved in the study	
☐	☒	Antibodies
☐	☒	Eukaryotic cell lines
☒	☐	Palaeontology and archaeology
☐	☒	Animals and other organisms
☒	☐	Human research participants
☒	☐	Clinical data
☒	☐	Dual use research of concern

Methods

n/a	Involved in the study	
☒	☐	ChIP-seq
☐	☒	Flow cytometry
☒	☐	MRI-based neuroimaging

Antibodies

Antibodies used	anti-KLRG1 (No. 46-5893; eBioscience/Affymetrix, Santa Clara, CA, USA), anti-CD127 (No. 17-1273; eBioscience/Affymetrix, Santa Clara, CA, USA), anti-CD8 (No. 48-0081; eBioscience/Affymetrix, Santa Clara, CA, USA), anti-CD4 (No. 11-0042; eBioscience/Affymetrix, Santa Clara, CA, USA), anti-CD3 (No. 25-0031; eBioscience/Affymetrix, Santa Clara, CA, USA), anti-CD44 (No. 11-0441; eBioscience/Affymetrix, Santa Clara, CA, USA), anti-CD62L (No. 83-062; eBioscience/Affymetrix, Santa Clara, CA, USA), anti-CCR7 (No. 47-1971; eBioscience/Affymetrix, Santa Clara, CA, USA), anti-CD45.1 (No. 47-0453; eBioscience/Affymetrix, Santa Clara, CA, USA), and anti-CD45.2 (No. 12-0454; eBioscience/Affymetrix, Santa Clara, CA, USA). anti-IFN-gamma (No. 17-7311; eBioscience/Affymetrix, Santa Clara, CA, USA), anti-TNF-alpha(No. 46-7321; eBioscience/Affymetrix, Santa Clara, CA, USA), and anti-IL-2 (No. 25-7021; eBioscience/Affymetrix, Santa Clara, CA, USA). AffiniPure goat anti-mouse IgG (H+L) (Jackson ImmunoResearch Laboratories Inc., West Grove, PA, USA)
Validation	The target specificities of antibodies purchased from eBioscience have been verified by the company.

Eukaryotic cell lines

Policy information about cell lines

Cell line source(s)	ATCC

Authentication	None of cell lines used in this study were authenticated.
Mycoplasma contamination	None of cell lines used in this study were tested for mycoplasma contamination.
Commonly misidentified lines (See ICLAC register)	No commonly misidentified lines were used in this study.

Animals and other organisms

Policy information about studies involving animals; ARRIVE guidelines recommended for reporting animal research

Laboratory animals	Female C57BL/6J, SCID, and B6.SJL-Ptprca Pepcb/BoyJ mice were obtained from Jackson Laboratory, Bar Harbor, ME, USA. IFNAR-/- mice were donated by Genhong Cheng at UCLA. Majority of animals in this study were between 6-12 weeks.
Wild animals	This study did not involve wild animals.
Field-collected samples	This study did not involve samples collected from the field.
Ethics oversight	The animal studies were approved by the Animal Research Committee at the University of California, Los Angeles (UCLA), Los Angeles, CA, USA.

Note that full information on the approval of the study protocol must also be provided in the manuscript.

Flow Cytometry

Plots

Confirm that:

- [ ] The axis labels state the marker and fluorochrome used (e.g. CD4-FITC).
- [ ] The axis scales are clearly visible. Include numbers along axes only for bottom left plot of group (a 'group' is an analysis of identical markers).
- [ ] All plots are contour plots with outliers or pseudocolor plots.
- [x] A numerical value for number of cells or percentage (with statistics) is provided.

Methodology

Sample preparation	Single-cell suspensions were obtained from the spleens and the red blood corpuscles were lysed in ACK (ammonium-chloride-potassium) buffer. Before staining, the splenocytes were incubated with FC block (No. 553142; BD Bioscience, Franklin Lakes, NJ, USA). Tetramers were incubated with splenocytes for 1 h at room temperature. Surface-staining with the following antibodies was performed by incubation at 4 °C for 30 min. For intracellular staining, BD Cytofix and Cytoperm (Cat. No. 554714; BD Bioscience, Franklin Lakes, NJ, USA) were used before staining.
Instrument	SORP BD LSRII analytic flow cytometer (BD Bioscience, Franklin Lakes, NJ, USA)
Software	FlowJo (FlowJo LLC, Ashland, OR, USA)
Cell population abundance	There was no sorting performed in this study.
Gating strategy	Cells for all flow cytometry experiments were gated on FSC-A/SSC-A to identify the cell population and to remove debris from the analysis. FSC-A/FSC-H and SSC-A/SSC-W were used to gate on single cells. Virus specific T cells were identified by gating on the CD8+Tetramer+ T cell population and were characterized by gating on CD127 and KLRG1. Functional T cells were assessed by gating on the CD45.2+ CD8+ population and characterized for the expression of IFN-γ, TNF-α, and IL-2. pDCs were identified by gating on the CD3 - CD19 - NK1.1 - B220 + CD11c Int PDCA-1 + population. Boundaries between positive and negative staining cell populations were defined using single fluorescence controls and fluorescence minus one (FMO) controls.

- [ ] Tick this box to confirm that a figure exemplifying the gating strategy is provided in the Supplementary Information.
